# Protocol for the Design and Assembly of a Light Sheet Light Field Microscope

**DOI:** 10.3390/mps2030056

**Published:** 2019-07-04

**Authors:** Jorge Madrid-Wolff, Manu Forero-Shelton

**Affiliations:** 1 Biomedical Computer Vision Group, Universidad de los Andes, Bogota 111711, Colombia; 2 Biophysics Laboratory, Universidad de los Andes, Bogota 111711, Colombia

**Keywords:** light field microscopy, light sheet fluorescence microscopy, LSFM, 3D imaging, fast imaging, open science

## Abstract

Light field microscopy is a recent development that makes it possible to obtain images of volumes with a single camera exposure, enabling studies of fast processes such as neural activity in zebrafish brains at high temporal resolution, at the expense of spatial resolution. Light sheet microscopy is also a recent method that reduces illumination intensity while increasing the signal-to-noise ratio with respect to confocal microscopes. While faster and gentler to samples than confocals for a similar resolution, light sheet microscopy is still slower than light field microscopy since it must collect volume slices sequentially. Nonetheless, the combination of the two methods, i.e., light field microscopes that have light sheet illumination, can help to improve the signal-to-noise ratio of light field microscopes and potentially improve their resolution. Building these microscopes requires much expertise, and the resources for doing so are limited. Here, we present a protocol to build a light field microscope with light sheet illumination. This protocol is also useful to build a light sheet microscope.

## 1. Introduction

Recent advances in fluorescent indicators such as calcium markers and transgenic model organisms have allowed the study of rapid dynamic biological processes in vivo like neural signaling, cardiovascular activity, and bacterial populations. Fast image acquisition methods have emerged in recent years to record these processes. Some of the fast methods use parts with little to no inertia in order to obtain slices at high speeds [1,2,3,4,5,6], while other methods such as extended depth of field [7,8] and integral imaging [9] capture the whole volume using a single camera exposure. Among these methods, Light Field Microscopy [10] allows for the fast acquisition of volumetric information of the sample from single camera exposures, at the expense of reduced spatial resolution. Nonetheless, light field microscopy has enabled functional imaging of neuronal activity at single-neuron resolution in the entire organism or portions of the larval zebrafish brain [11,12,13], adult *Drosophila melanogaster* [14], and mammalian brains [15].

This up-and-coming technique requires custom-made optical setups, which are intricate and non-trivial to design and align. Here we show a protocol to build and align such a microscope and include some basic design guidelines.

## 2. Experimental Design

The major difference between a conventional microscope and a light field microscope is the inclusion of microlenses, which must be matched to the objective lens in f-number [16], making objective interchanges cumbersome for a single experiment. In this setup we have included a second detection arm to help with sample navigation, which is one of the main reasons why objectives are changed during an experiment. This arm has a shorter tube lens that results in a lower magnification and a larger field of view that facilitates finding the area of interest in a sample, or the sample itself. We also use this portion of the detection to align the system.

In the context of light field this is particularly important since it is hard to interpret light field data by eye, as it requires additional software or hardware and is not usually done in real time, although methods have been demonstrated [17].

In order to minimize light losses and maintain signal-to-noise ratios, the light sheet detection arm uses the long-wavelength tail of GFP´s emission spectrum for detection. A dichroic (Semrock, Rochester, NY, USA, FF560-FDi02) splits the light field from the light sheet detection arms around 560 nm. A bandpass (Chroma, Bellows Falls, VT, USA, ET525/50 m) filters the GFP signal for the light field, and a second bandpass between (Chroma, Bellows Falls, VT, USA, ET575/50 m) filters the light for the light sheet detection. Alternatively, a 90–10 beam splitter (Thorlabs, Newton, NJ, USA, BS076) and a single GFP fluorescence filter may be used.

Several aspects should be considered in order to design the detection arm of a light field system. First, the f-numbers of the objective and tube lens combination must be matched. Mismatch will result in overlapping of the microlenses’ projections on the camera chip or sub-optimal use of the number of pixels. If the f-number of the detection system is smaller than that of the microlenses there will be light rays that will cross over from one microlens’ detection pixels to another’s, significantly compromising the reconstruction. Conversely, if the microlenses have a much smaller f-number than the detection system, there will be an increased number of pixels to which no light arrives. This will compromise the axial resolution of the system as less pixels will be available for sampling the impinging light rays. The f-numbers of microscope objectives are relatively standard, so matching is done by selecting from off-the-shelf microlens arrays (which come in a variety of f-numbers), and, if necessary, by adjusting the f-number of the objective-tube lens system. This is done by using a tube lens of a different focal length than the objective’s standard. Although this will influence image quality, changes in the order of 50% are manageable. Using a tube lens of a different focal length will result in a modified effective magnification, affecting the final resolution of the system. Second, we consider the desired resolution. Lateral resolution of the resulting reconstructions will be given by the number of microlenses in the array. Axial resolution depends on the number of resolvable spots on the camera chip behind each lenslet, as described by Levoy et al. [10]. Using a camera with a high pixel density is recommended to adequately sample the incoming rays.

Recently, more complex methods such as compressive sensing [18], multiplexing [19], and speckle-based structured illumination [20] have further improved the axial resolution in light field microscopy imaging.

Then one should consider the design of the illumination. The height of light sheets should be comparable to the field of view (FoV) of the detection, which can be calculated as

(1) $$FoV=\frac{Camera\;chip\;size}{M}\;$$

where *M* is the magnification of the detection. Thus, the height of the light sheet (its extent along the *y*-axis, may be computed in terms of the original beam width and the lenses’ focal lengths as

(2) $$height_{light\;sheet}=w_{out}\;\frac{f_{beam\;expander\;._{long}}}{f_{beam\;expander_{short}}}\;\frac{f_{scan\;lens}}{f_{cylindrical\;lens}}\;\frac{f_{ill.\;obj.}}{f_{tube\;lens}}\;$$

where $w_{out}$ is the beam radius at the output of the laser (typically ~500 μm). Note that the width of the beam at the galvanometric mirror

(3) $$2w_{galv.}=2w_{out}\;\frac{f_{beam\;expander\;._{long}}}{f_{beam\;expander_{short}}}\;$$

may not exceed the size of the mirror, otherwise the beam will be clipped. The width of the light sheet at its waist depends on the effective numerical aperture of the illumination:

(4) $$NA_{eff.}=NA\frac{2w_{out}\;\frac{f_{b.e._{long}}}{f_{b.e._{short}}}\frac{f_{tube\;lens}}{f_{scan\;lens}}}{Pupil\;width_{ill.\;obj.}}\;$$

where $Pupil\;width_{ill.\;obj.}$ is the size of the back pupil of the illumination objective. If the fraction right to *NA* is greater than 1, some clipping of the beam will occur at the pupil, which is manageable. The width of the light sheet at its waist will be given by, and also its Rayleigh length (the distance at which its width will have doubled) by

(5) $$2w_{0}=\;\frac{\lambda }{2NA_{eff.}}\;$$

(6) $$x_{R}=\;\frac{\pi w_{o}^{2}}{\lambda }\;$$

where *λ* is the laser’s wavelength. Equation (6) may be used to compute the extent of the light sheet along the optical axis of the detection. As the light sheet widens, contrast decreases and optical sectioning becomes less effective. The Rayleigh length of the light sheet should be matched with the size of the sample and the FoV of the detection.

**Figure 1:**
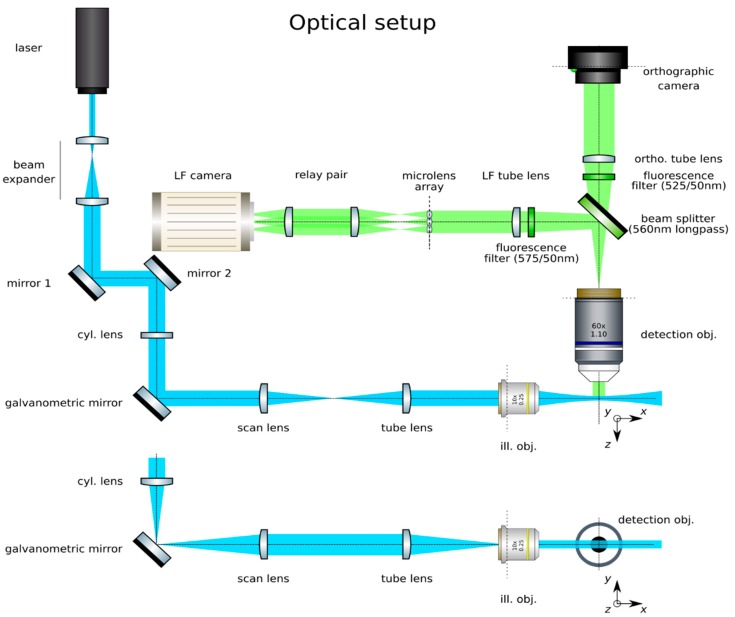
Optical setup of a light sheet light field microscope.

In our proposed setup, shown in Figure 1, we have a beam which is 4 mm at the galvanometric mirror and slightly overfills the 12 mm wide back pupil of the illumination objective along the *z*-axis with an extension of 13.2 mm to produce light sheets which are 350 μm high and, at best, 1 μm at their waist. The FoV of the LF detection at $M_{eff.}=50$× is 260 μm × 260 μm and 205 μm × 164 μm for the orthographic detection. Olarte et al. provide more comprehensive details on the design of a light sheet microscope [21].

### 2.1. Materials

- Silver protected mirrors (Thorlabs, USA, PF10-03-P01)
- Kinematic mounts (Thorlabs, USA, KM100)
- Aperture irises (Thorlabs, USA, ID20)
- f = 7.5 mm achromat (Thorlabs, USA, AC050-008-A-ML)
- f = 40 mm achromat (Edmund Optics, Barrington, NJ, USA, 49–354)
- 1” lens mount (Thorlabs, USA, LMR1/M)
- 0.5” les mount (Thorlabs, USA, SMR05/M)
- f = 65 mm f-θ lens (Sill Optics, Wendelstein, Germany, S4LFT0061/065)
- f = 250 mm achromat (Edmund Optics, USA, 49–366)
- f = 75 mm mounted achromatic cylindrical lens (Thorlabs, USA, LJ1703RM)
- N PLAN 10x NA 0.25 dry objective (Leica Microsystems, Wetzlar, Germany)
- f = 100 mm achromat (Edmund Optics, USA, 49–360)
- Cage plate (Thorlabs, USA, CP02/M)
- SM1 coupler (Thorlabs, USA, SM1T2)
- SM1 to C-mount adapter (Thorlabs, USA, SM1A9)
- 4” cage rods (Thorlabs, USA, ER4-P4)
- Dichroic beam splitter (Semrock, USA, FF560-FDi02)
- Cage cube (Thorlabs, USA, CM1-DCH/M)
- Fluorescence band-pass filter for GFP (Chroma, USA, ET525/50 m)
- Fluorescence band-pass filter for GFP (Chroma, USA, ET575/50 m)
- LUMFLN 60x NA 1.1 water immersion objective (Olympus, Tokyo, Japan, LUMFLN 60XW)
- f = 150 mm achromat (Edmund Optics, USA, 49–362)
- 1:1 Relay pair (Thorlabs, USA, MAP10100100-A)
- Lens tube for 1” optics (Thorlabs, USA, SM30L05)
- Microlense array (RPC, USA, MLA-S100-f21)
- 6-axis kinematic mount (Thorlabs, USA, K6XS)

### 2.2. Equipment

- 488 nm CW diode laser (Oxxius, Lannion, France, LBX-488-50-CSB-PP)
- Galvanometric mirrors (Edmund Optics, USA, 6215H)
- CCD monochrome camera (Point Grey, BC, Canada, Black Fly 13H2M)
- Micromanipulator (Sutter Instruments, Novato, CA, USA, MPC-200)
- sCMOS camera (Hamamatsu, Japan, ORCA flash 4.0 V2)
- xyz kinematic mount (Newport, RI, USA, 9063-XYZ-M)

## 3. Procedure

### 3.1. Construction of Light Sheet Illumination

Time for completion: 12 h (may be interrupted at any point). This section deals with the construction and alignment of a single-wavelength scanned-light sheet illumination. The light sheet will be a Gaussian beam produced by a cylindrical lens scanned by a galvanometric mirror.

**Figure 2:**
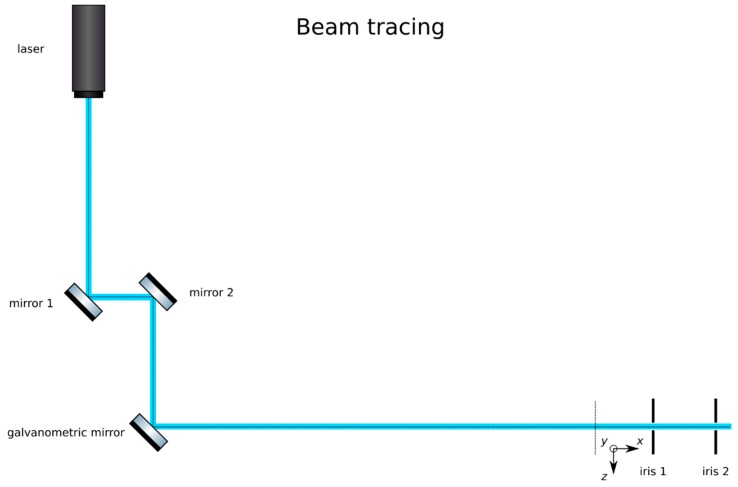
Tracing the beam path with a pair of mirrors, the galvanometric mirror. Record the path with a pair of irises.

**Figure 3:**
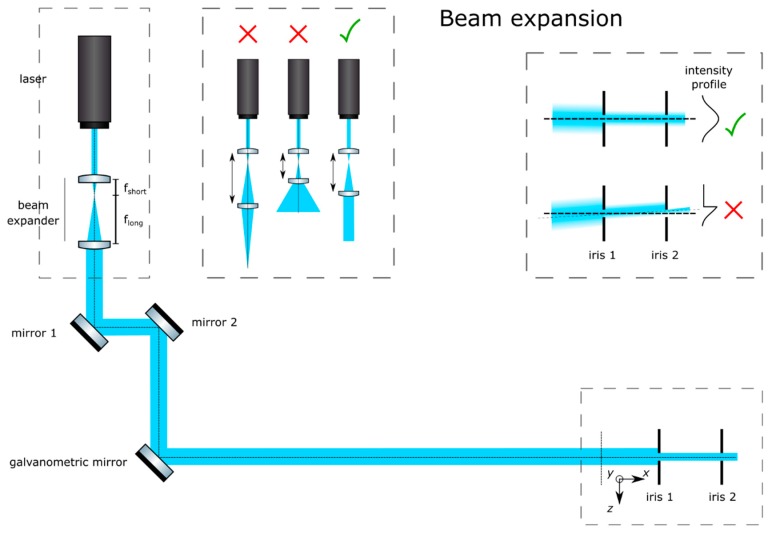
Beam expansion. Adjust the distance between the two lenses to guarantee that a collimated beam is achieved. Adjust their lateral positions so that the beam is not deviated.

**Figure 4:**
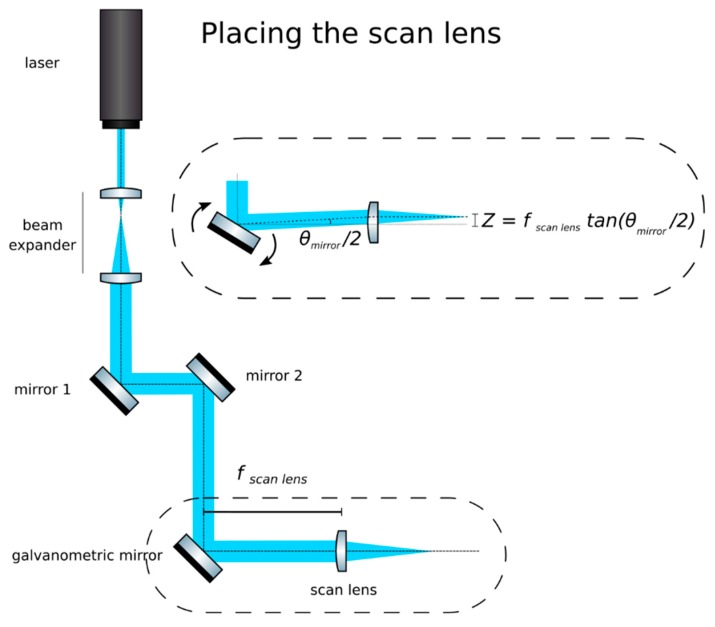
The scan lens should be one focal distance away from the galvanometric mirror. If placed correctly, rotations of the mirror will produce displacements but not rotations of the beam.

**Figure 5:**
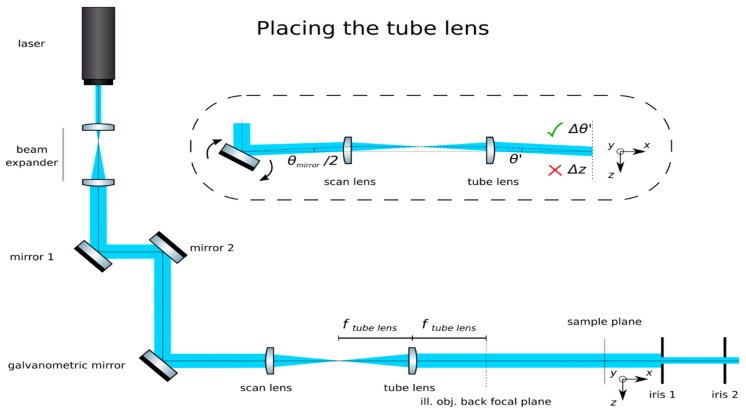
Placing the tube lens. The tube lens should produce a beam expander with the scan lens. If placed correctly, a well collimated beam will be achieved and, at the its focal plane, which will coincide with the illumination objective’s back focal plane, rotations of the mirror will produce rotations (Δθ), but not translations (Δz) of the beam.

**Figure 6:**
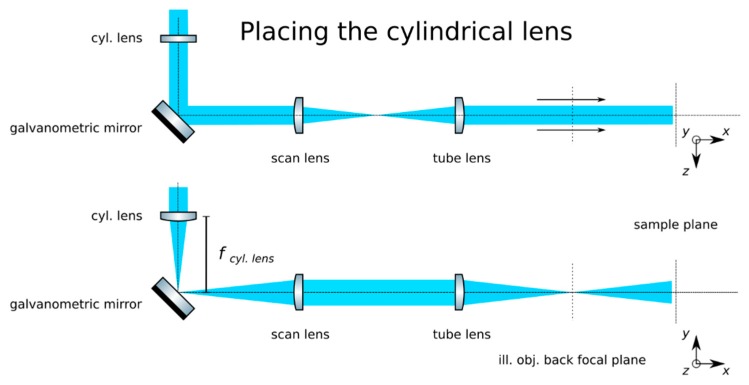
Place the cylindrical lens before the galvanometric mirror. Ensure that its focal plane lies on the mirror’s surface.

**Figure 7:**
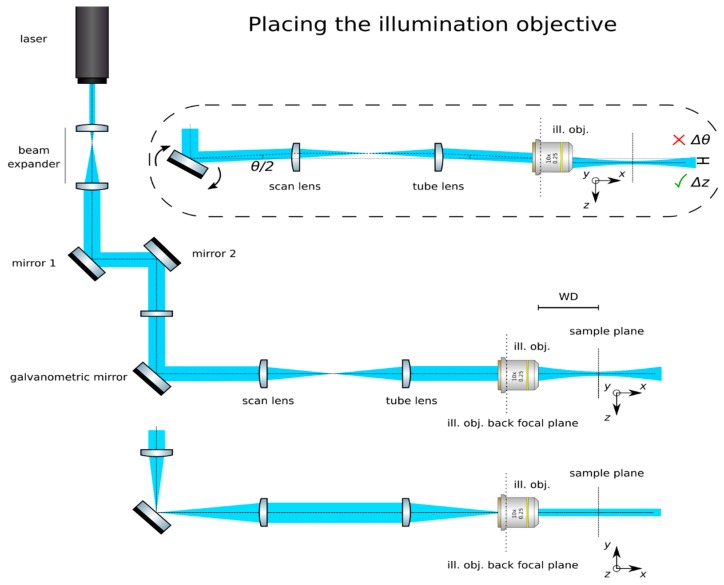
Placing the illumination objective. If placed correctly, a collimated beam should be achieved along the *y*-axis, while strongly converging into a light sheet along the *z*-axis.

1. **Trace the beam path**: Trace the beam path from the laser (Oxxius, LBX-488-50-CSB-PP) to the place where the sample will go by means of mirrors (Thorlabs, PF10-03-P01) in kinematic mounts (Thorlabs, KM100), as in Figure 2. Place the galvanometric mirror (Edmund Optics, 6215H) in the path. Mark the beam path at its end (ideally downstream from the sample’s position) with a pair of irises (Thorlabs, ID20). Do not change the position of the irises afterwards.
2. **Expand the beam**: Expand the laser beam as shown in Figure 3. Mount a pair of lenses, one of short negative focal length (Thorlabs, f = 7.5 mm, AC050-008-A-ML) and a second lens of positive long focal length (Edmund Optics, f = 40 mm, 49–354) in lens mounts (Thorlabs, SMR05/M and LMR1/M, respectively). First, insert the short lens in the beam path. Finely adjust the position and orientation of this lens to make sure that the center of the beam still passes through the center of the irises. Insert the lens of larger focal length approximately 47.5 mm (the sum of the focal lengths) downstream from the first lens. Finely adjust its position and orientation to make sure that the center of the beam still passes through the center of the irises. Alternatively, cage plates (Thorlabs, CP02/M) and cage rods (Thorlabs, ER3-P4) may be used to ensure that the lenses are aligned along the optical axis. Adjust its position along the optical axis to ensure that an expanded collimated beam is achieved. This may be verified by measuring the width of the beam at two points downstream and ensuring that it remains constant by focusing the beam at infinity (i.e., several meters away), or with the aid of a shearing interferometer (Thorlabs, SI050).
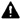
**CRITICAL STEP: Insert the scan lens:** Place the scan lens (We use an f-θ lens as scan lens. A regular achromat may be used as well). (Sill Optics, S4LFT0061/065) a focal length downstream from the galvanometric mirror, as in Figure 4. Finely adjust its position to ensure that rotations of the mirror produce translations of the beam from the main optical axis while remaining parallel to it. Additionally, check that translations correspond to rotations following the equation
  (7) $$z=f_{scan\;lens}\tan\left(\frac{\theta_{mirror}}{2}\right)\;$$
4. **Optional Step: Produce a calibration function for the galvanometric mirror:** The controlling software of some galvanometric mirrors often indicate the voltage applied to the device, but not the induced angle of rotation. Manufacturers should include the relationship θ(V) in their documentation. Collect data for a beam displacement vs. voltage calibration function, namely *z(V).*
5. **Insert the tube lens**: Place the tube lens (Edmund Optics, f = 250 mm, 49–366) and adjust its position until a collimated beam is obtained, as in Figure 5.
6. **Insert the cylindrical lens:** Place the cylindrical lens (Thorlabs, f = 75mm, LJ1703RM) and adjust its position along the optical axis until a sharp focused line appears precisely on the galvanometric mirror, as in Figure 6. Galvanometric mirrors are very small; check that the beam is not clipped by the mirror. The orientation of the lens matters: place its flat face pointing toward its focus and ensure that a line along the *z*-axis will be produced at the back pupil of the illumination objective.
7. **Check the alignment**: The focused line formed by the cylindrical lens should be relayed by the scan and tube lenses onto the back focal plane of the microscope objective. Check that while rotating the galvanometric mirror, the light sheet rotates but is not displaced at this plane.
8. **Insert the illumination objective**: Place the illumination microscope objective (Leica N PLAN 10x NA 0.25). Adjust its position along the optical axis until a beam which is collimated along the *y-*axis but highly divergent along the *z*-axis is achieved, as in Figure 7.

### 3.2. Construction of the Orthographic Detection 

Time for completion: 3 h

**Figure 8:**
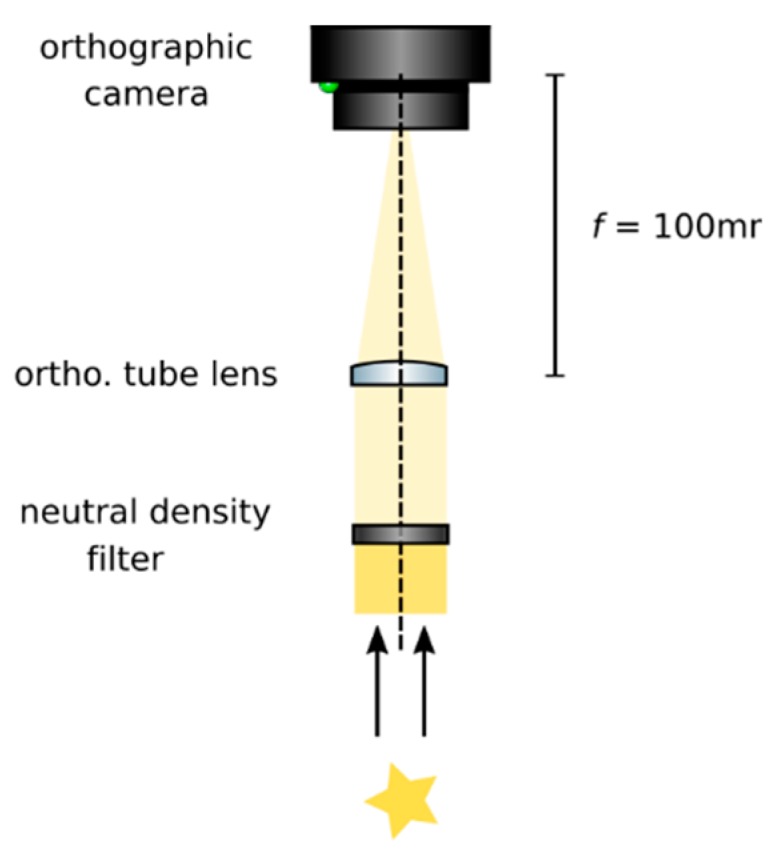
Building an orthographic detection. Adjust the distance between the tube lens and the camera until a sharp image of a distant object is achieved, or a collimated beam appears focused. If necessary, use a neutral intensity filter to reduce the intensity of light reaching the camera to avoid damaging it.

**Figure 9:**
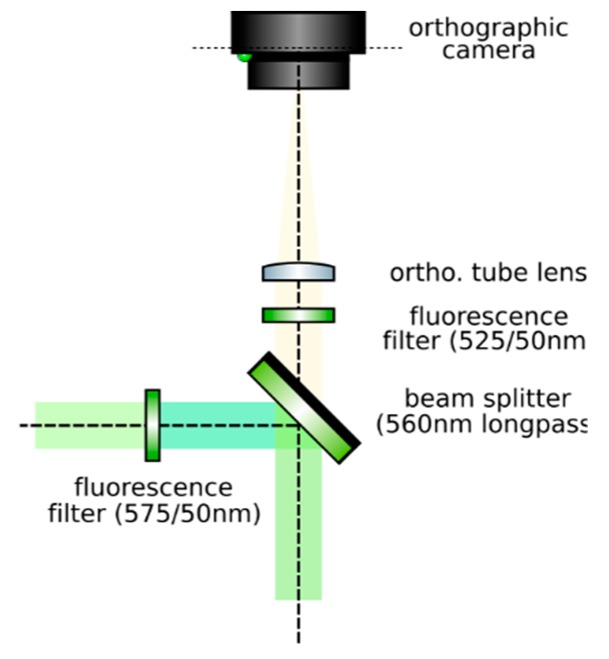
Insert a beam splitter and two fluorescence filters before the tube lens.

**Figure 10:**
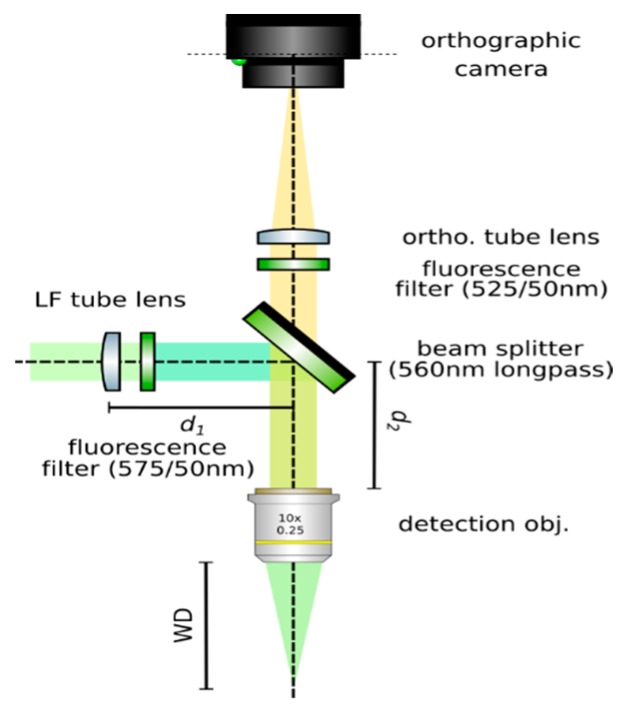
Place a detection objective.

**Figure 11:**
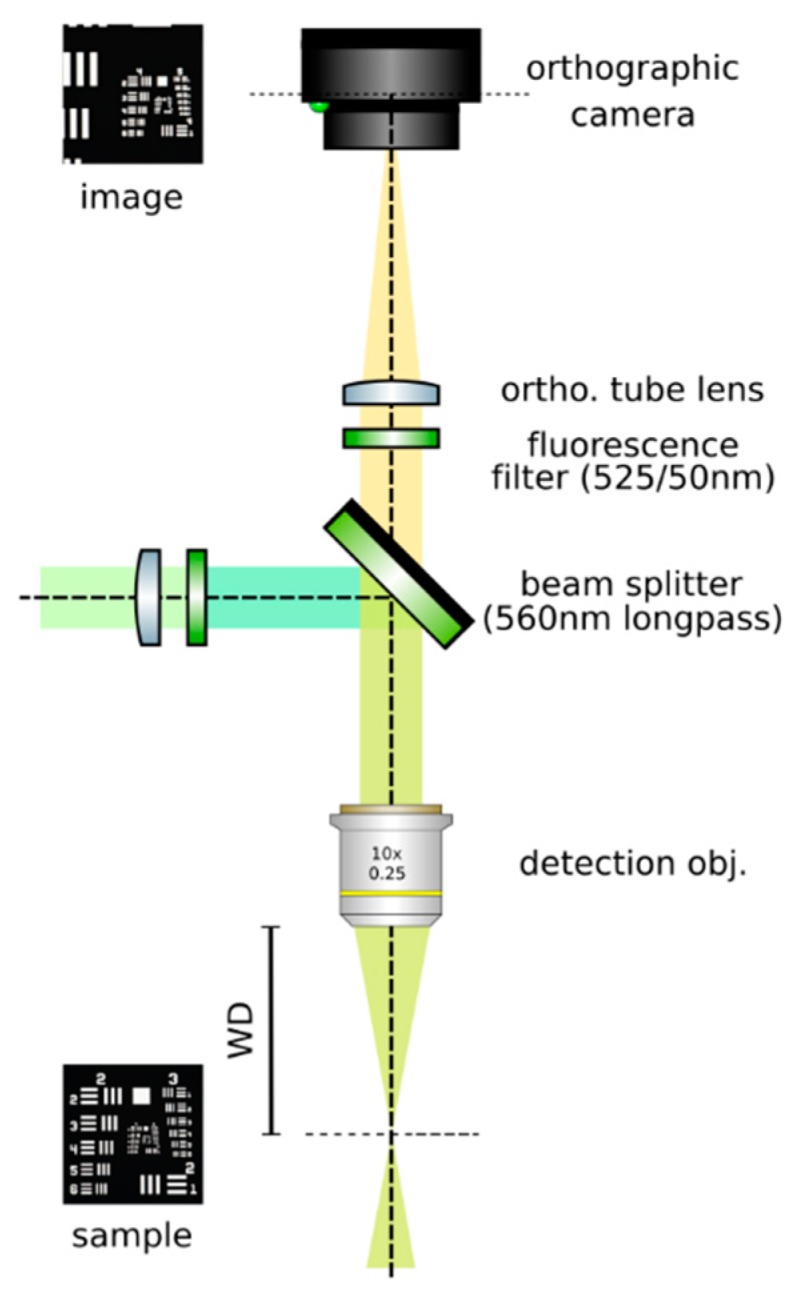
Image a high-contrast sample. Displace the sample until a sharp image of it appears on the orthographic camera.

1. **Set up an orthographic detection system:** Mount an achromatic tube lens (Edmund Optics, f = 100 mm, 49–360) in a cage plate (Thorlabs, CP02/M). Attach an inspection camera (Point Grey, Black Fly 13H2M) to a cage plate by means of a SM1 coupler (Thorlabs, SM1T2) and a SM1 to C-mount adapter (Thorlabs, SM1A9). Assemble the cage system with the use of four rods (Thorlabs, ER4). Adjust the distance from the tube lens to the camera sensor by obtaining a point-like image of a collimated light beam, or alternatively, by imaging a distant object, as in Figure 8.
2. **Insert the beamsplitter:** Mount a dichoric beamsplitter (Semrock, FF560-FDi02) in a cage cube (Thorlabs, CM1-DCH/M). Attach the cube to the cage system built on step 1 so that the transmitted light from the beamsplitter reaches the inspection camera. Mind the orientation of the beamsplitter (refer to the manufacturer’s documentation for this).
3. **Insert two fluorescence filters:** Mount a fluorescence band-pass filter (Chroma, ET525/50 m) in a cage plate. Attach the cage plate to the cage cube from step 2 by means of cage rods. Light reflected from the dichroic filter should then go through the bandpass filter. Mind the orientation of the bandpass filter. Mount a second fluorescence band-pass filter (Chroma, USA, ET575/50 m), this time for the inspection camera. See Figure 9.
4. **Insert a detection objective:** Mount an infinity-corrected microscope objective 140–170 mm in front of the achromatic tube lens to a cage plate, as in Figure 10. Use a dry long-working distance objective for microscope construction, even if the apparatus is designed to work with water immersion objectives. We use a Leica 10× NA 0.25 air objective with external M25 threads that we couple via a thread adapter (Thorlabs, SM1A12) to a cage plate. Attach the cage plate to the two by means of cage rods.
5. **Use homogeneous high-NA illumination:** Place a Köhler Illumination module in front of the objective. Maximize the NA of the illumination to reduce the depth of field of the system and thus ensure better positioning of the light field camera in the upcoming steps.
6. **Focus a high contrast sample:** Place a flat, high-contrast sample (ideally of known size) and obtain a sharp image of it on the inspection camera by adjusting its distance to the microscope objective, as in Figure 11. A micromanipulator (Sutter Instruments, MPC-200) is useful for this task. Keep the sample in this fixed position.

### 3.3. Construction of the Light Field Detection

**Figure 12:**
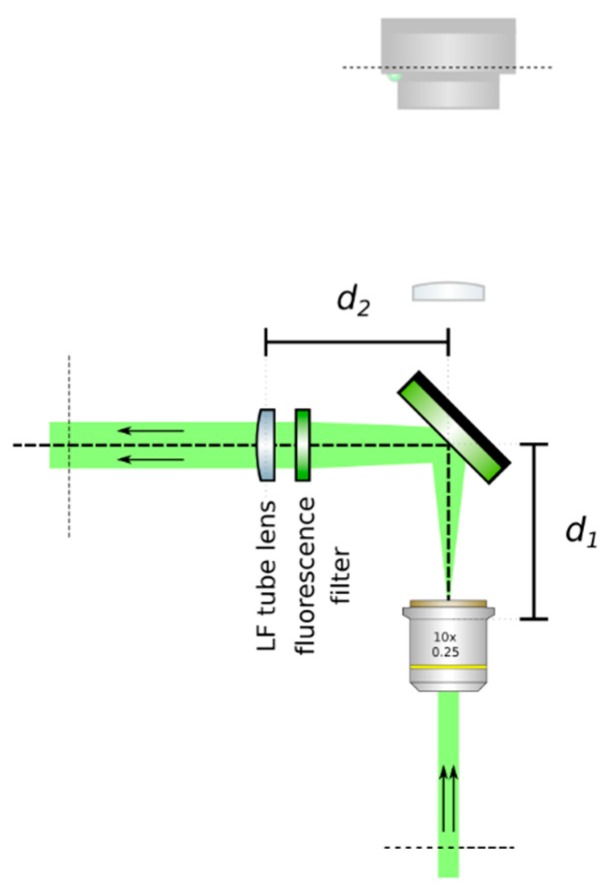
Insert the LF tube lens. Using collimated light, adjust the distance from the detection objective to the LF tube lens until a collimated beam results from the tube lens. This guarantees a 4*f* configuration between the objective and the tube lens.

**Figure 13:**
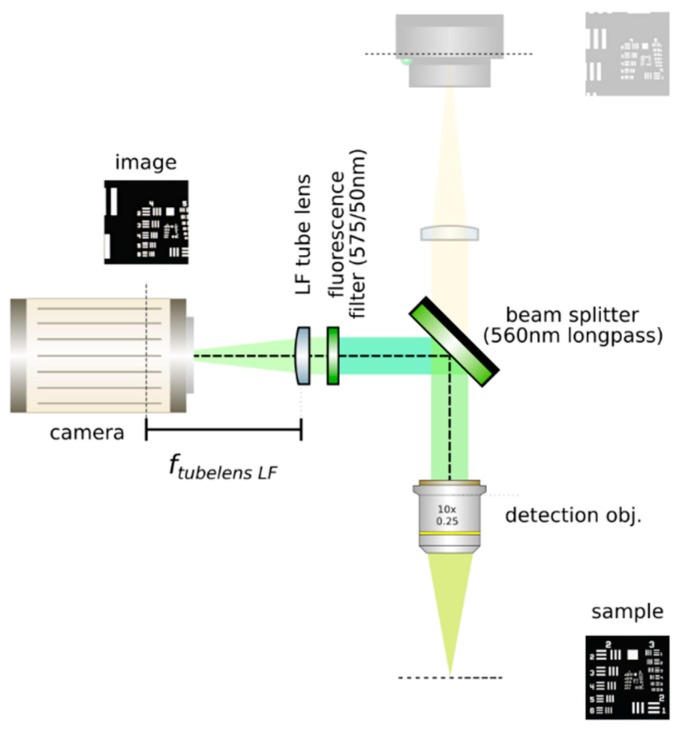
Place a second camera and acquire a reference image. Place a camera after the LF tube lens, making sure to obtain a sharp image of the same portion of the sample such as that seen on the orthographic camera. Do not displace the sample. Acquire a reference image and measure the sample. Use this image to ensure magnification when inserting the relay pair.

**Figure 14:**
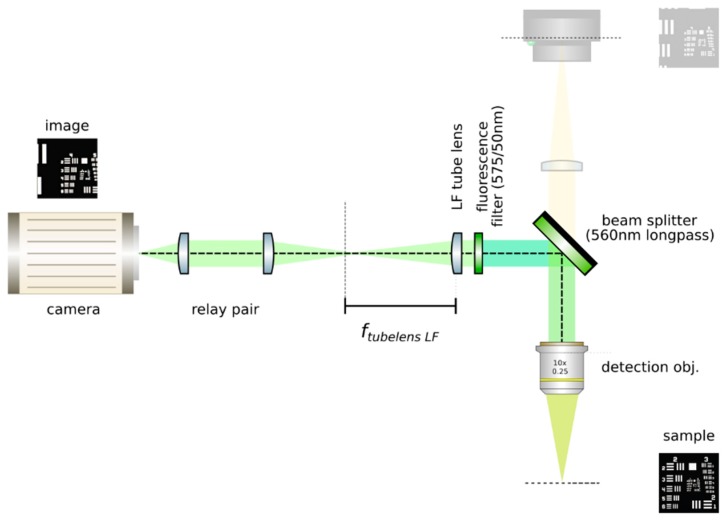
Insert a relay pair. Move the camera backwards and place the relay pair. An image of the sample should appear in focus with the correct magnification (from the relay).

**Figure 15:**
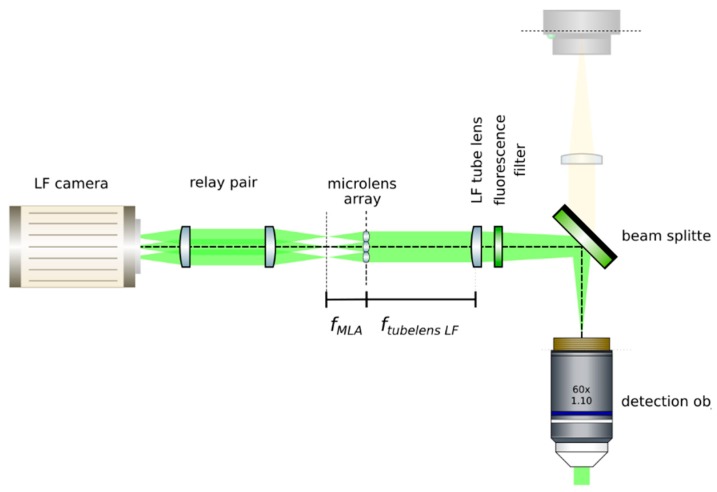
Insert the microlens array at the image plane of the tube lens. This is ensured when an image of the microlenses appears superimposed on the image of the sample. Then, using paraxial illumination, jointly move the relay pair and the camera until a grid of dots appears on the sensor. This ensures that the back focal plane of the lenslet array is imaged.

**Figure 16:**
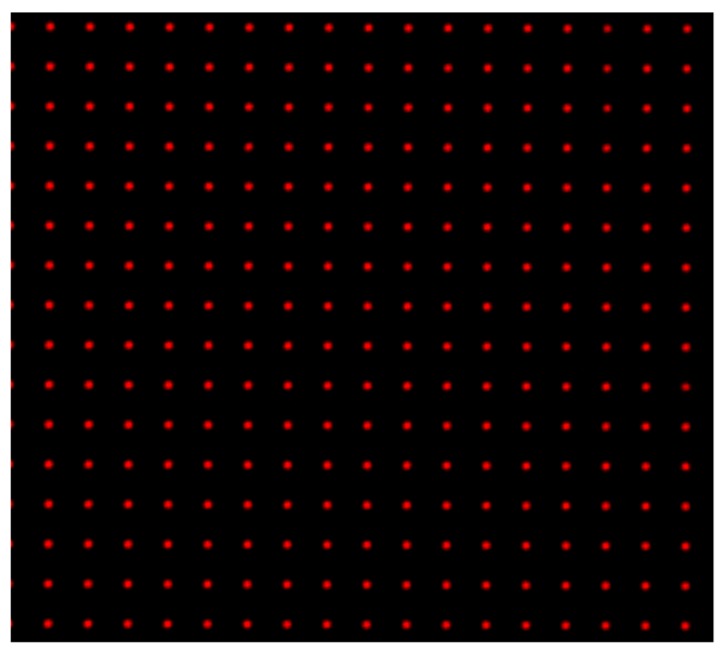
Back focal plane of the microlenses. When imaging the back focal plane, a grid of dots will appear in the camera chip. Each dot should be located at the center of each microlens. Use the 6-axis kinematic mount to ensure that the yaw and pitch angles of the microlens array are correct (if they are not, some microlenses will be in focus, while others are not). Ensure that the microlens grid is as aligned with the pixel grid as possible. (Magnified view).

**Figure 17:**
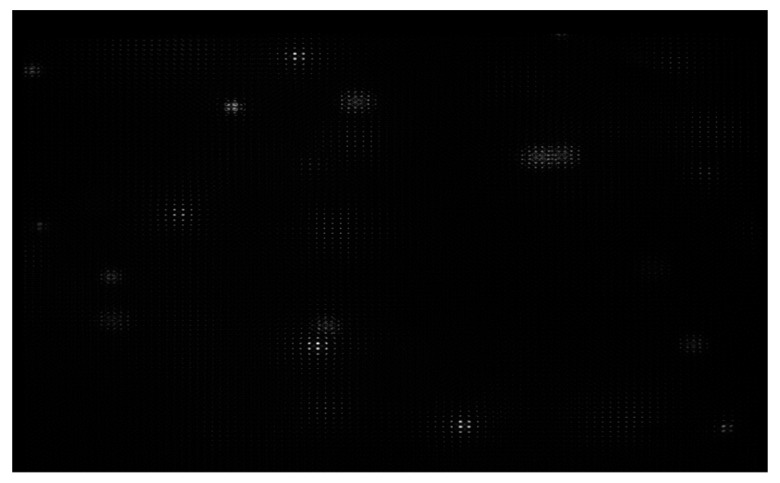
Registered light field of 6μm fluorescent beads in agar. The light field should appear as a grid of non-overlapping circles.

Time for completion: 10 h

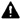
**CRITICAL STEP: Insert the LF tube lens**: Mount the main tube lens, an f = 150 mm achromat (Edmund Optics, 49–362), in a cage plate. Insert this cage plate in the cage rods from step 3, after the bandpass filter. The light field system is an afocal detection system, and thus, the detection objective and the LF tube lens must form a 4f system, as in Figure 12; that is, the back focal plane of the detection objective and the front focal plane of the main tube lens must coincide. To achieve this, send a collimated beam through the objective and adjust the position of the LF tube lens until a collimated (although expanded) beam exits it.
2. **Place a second camera:** Attach a second camera that will record the light fields (Hamamatsu, ORCA flash 4.0 V2) to an xyz kinematic mount (Newport, 9063-XYZ-M), as in Figure 13. Place the camera downstream from the LF tube lens. Move it until a centered sharp image of the target appears in focus. While inspecting both cameras simultaneously, adjust the lateral and vertical position of the LF camera until both images are centered in the same place of the sample (one will be reflected by the beam splitter). Fix the camera to this position.
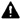
**CRITICAL STEP: Acquire a reference image:** Acquire an image of the sample at this moment. Measure it using image analysis software, such as ImageJ [22]. This image is important to guarantee that the right magnification is achieved when a relay pair of lenses is inserted in the upcoming steps. Keep the sample at its position until indicated. Do not pause the protocol until the end of step 4. The position of the sample may be lost, and magnification of the system may not be guaranteed.
4. **Insert a relay pair:** Move the main camera away from the main tube lens in order to give space to a relay lens pair. Insert the relay pair (Thorlabs, MAP10100100-A) in the light field detection; see Figure 14. Ideally, it should be mounted so that it may be moved jointly with the main camera, as by attaching it to a lens tube (Thorlabs, SM30L05). The relay pair is needed to project the back focal plane of the microlenses onto the LF camera’s chip, as microlenses have a very short focal length. Adjust the positions of the camera and the relay pair until a sharp image with the desired magnification (that introduced by the relay pair) is achieved. Calculate the magnification by measuring the size of the sample’s image and comparing it with the one obtained in step 3.
5. **Insert the microlens array:** Mount a microlens array (RPC, MLA-S100-f21) in a 6-axis kinematic mount (Thorlabs, K6XS). Insert the microlens array at the image plane of the LF tube lens, as in Figure 15. The microlenses will sit on the image plane of the main tube lens when a sharp image of their edges appears on top of the sample. Adjust the pitch and yaw angles of the microlens array by means of the 6-axis kinematic mount until the central region of the microlens array is uniformly in focus. Rotate the microlens array to have it as aligned as possible with the pixel grid of the camera. Move the camera backwards and place the relay pair. An image of the sample should appear in focus with the correct magnification (from the relay).
6. **OPTIONAL STEP:** Remove the sample from the field of view.
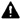
**CRITICAL STEP: Image the back focal plane of the microlenses:** Provide paraxial illumination, as in Figure 15. This may be done by closing both the field and aperture diaphragm of the Köhler illumination system or by sending an expanded well-collimated beam through the detection.
8. Move the relay pair and the main camera away from the microlens array by its back focal length. Image the back focal plane of the microlenses. This will be achieved when a grid of the smallest spots is visible, such as in Figure 16. If possible, use light of a long wavelength to maximize the effects of diffraction on the lenslet array [23].
9. **Place the high NA detection objective:** Put the high magnification, high NA objective (Olympus, LUMFLN 60XW) in the detection path. We use a custom 3D printed chamber to place the sample. Record a light field, such as the one in Figure 17.

## 4. Expected Results

**Figure 18:**
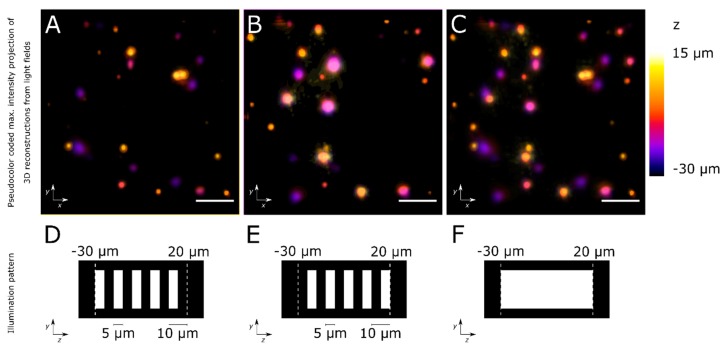
Reconstructions of light fields of 6μm fluorescent beads in agar. (**A**,**B**) Reconstructions of light fields from complementarily illuminated portions of the sample volume. (**C**) Reconstruction of the light field from the volume under bright field illumination. (**D**–**F**) Profiles of illumination patterns for (**A**–**C**), respectively. In (**D**,**E**), sets of 5 μm wide light sheets provide optical sectioning of the volume. Scalebars 50 μm.

To produce 3D imagery from light fields, such as those illustrated in Figure 18, there is open software for reconstructions [10,11,17,23,24]. In our reconstructions, we show that by using light sheets to selectively illuminate the sample’s volume, we may separate features in complementary acquisitions, which may in turn ease localization and improve resolution.
